# Supplementary material for: The genome sequence of the Small Sallow Mining Bee, Andrena praecox (Scopoli, 1763)
Source: Wellcome Open Res. 2025 Jun 2;10:290. [Version 1] doi: 10.12688/wellcomeopenres.24301.1 (PMC12344408; doi:10.12688/wellcomeopenres.24301.1)
Supplement: Peer review history

# Reviewer report: Reviewer response for version 1

**DOI:** 10.21956/wellcomeopenres.26800.r126862

**Published:** 2025-08-23

**Authors:** Rodolpho Menezes

**Affiliations:** 1 State University of Santa Cruz, Ilhéus, Brazil

The manuscript by Crowley presents a genome assembly from a female *Andrena praecox* (Scopoli, 1763), resulting in a final assembly size of 563 megabases. This assembly represents a valuable resource for comparative genomics, as well as for studies on bee evolution and conservation. The methodology is thorough and clearly described, the figures are well constructed, and the manuscript is generally well written.

 However, I recommend that the author compare this assembly with those of other bee species to contextualize the results more effectively. Additionally, it would be valuable to include an analysis of repetitive elements, including the proportion of the genome they occupy and the types of repeats that are most prevalent.

Are sufficient details of methods and materials provided to allow replication by others?

Yes

Is the rationale for creating the dataset(s) clearly described?

Yes

Are the datasets clearly presented in a useable and accessible format?

Yes

Are the protocols appropriate and is the work technically sound?

Yes

Reviewer Expertise:

Cytogenetics, Phylogeography and Phylogenomics

I confirm that I have read this submission and believe that I have an appropriate level of expertise to confirm that it is of an acceptable scientific standard.

# Reviewer report: Reviewer response for version 1

**DOI:** 10.21956/wellcomeopenres.26800.r126858

**Published:** 2025-08-19

**Authors:** Paulo Cseri Ricardo

**Affiliations:** 1 University of Sao Paulo, São Paulo, Brazil

This article presents data on the genome assembly of the bee species *Andrena praecox*. A robust approach was used to generate chromosome-level scaffolds, combining PacBio HiFi long reads with Hi-C mapping. Assembly metrics indicated high quality (QV) and completeness (k-mer and BUSCO). Most protocols were clearly described or appropriately referenced to ensure reproducibility. The final assembly spans 563.05 Mb across 664 scaffolds, with seven scaffolds accounting for 78.66% of the total sequence (chromosomal pseudomolecules), in addition to a 20.36 Kb mitogenome. Notably, a portion of the assembly did not align with the pseudomolecules and, for the most part, lacked hits to known taxa in BlobToolKit analyses. Examining these smaller scaffolds could clarify whether they stem from contaminants, low-complexity sequences, or assembly artifacts. Overall, the manuscript is clearly and objectively written and presents relevant data for studies on hymenopteran genome evolution, particularly in bees. I consider it suitable for indexing in its current form.

Are sufficient details of methods and materials provided to allow replication by others?

Yes

Is the rationale for creating the dataset(s) clearly described?

Yes

Are the datasets clearly presented in a useable and accessible format?

Yes

Are the protocols appropriate and is the work technically sound?

Yes

Reviewer Expertise:

Transcriptomics and genomics applied to the evolution and molecular ecology of bee species.

I confirm that I have read this submission and believe that I have an appropriate level of expertise to confirm that it is of an acceptable scientific standard.

# Reviewer report: Reviewer response for version 1

**DOI:** 10.21956/wellcomeopenres.26800.r126861

**Published:** 2025-08-12

**Authors:** Jason L. Williams

**Affiliations:** 1 University of Florida, Florida, USA

In this manuscript, the authors present a chromosomally complete genome sequence of the small sallow mining bee, *Andrena praecox*, supported by detailed sequencing, assembly statistics, and thorough quality assessment and annotation. This high-quality genome represents a valuable resource for investigating the evolution, ecology, and conservation of wild pollinators and provides a solid foundation for comparative genomics within *Andrena* and across Hymenoptera.

 Overall, the assembly is supported by rigorous methods and demonstrates strong completeness and accuracy, as evidenced by high contiguity, base-level quality, and robust BUSCO and k-mer metrics. The annotated gene set enhances the utility of the genome for downstream analyses, and the authors ensure transparency and reusability by making all data openly accessible.

 Despite the overall high quality of the genome assembly, a few areas could benefit from clarification or future improvement. These include slightly sub-threshold k-mer completeness in the primary assembly, incomplete chromosomal assignment, absence of identifiable sex chromosomes, and lack of functional annotation summary.

 Suggestions for improvement:

- Notably, there is no discussion section in the manuscript. Perhaps one could be included to offer further clarification of some points laid out below.
- Although combined k-mer completeness is over 96%, the primary assembly on its own only accounts for 90.56%. This is not an issue, but it might be worth elaborating in the manuscript on what genomic regions (e.g., repetitive sequences, heterochromatic regions, etc.) might be under-represented or missing and why.
- Only 78.66% of the genome is scaffolded into chromosomes, well below the 90% benchmark cited in Table 2. Have any additional attempts been made to help reach the benchmark? Would additional Hi-C rounds or linkage mapping help close this gap? At the very least, some explanation about this gap in scaffolding completeness is warranted.
- The assembly lacks identification of sex chromosomes, which are biologically important in bees and other Hymenoptera due to haplodiploid sex determination. It is not uncommon to face difficulty in identifying sex chromosomes of Hymenoptera, but the authors should briefly discuss this point and provide some interpretation as to why.
- While gene annotation is included, no summary of functional categories (e.g., GO terms or pathways) is provided. Including or referencing such data would increase the genome’s utility for downstream analyses.
- Finally, the manuscript would benefit from a brief explanation of the practical significance of sequencing *Andrena praecox*. Clarifying why this species was selected beyond the broad goal of sequencing all named eukaryotes in the Atlantic Archipelago and highlighting potential applications in ecology, conservation, or pollination biology would enhance the work’s impact and relevance.

Are sufficient details of methods and materials provided to allow replication by others?

Yes

Is the rationale for creating the dataset(s) clearly described?

Partly

Are the datasets clearly presented in a useable and accessible format?

Yes

Are the protocols appropriate and is the work technically sound?

Yes

Reviewer Expertise:

Insect biodiversity, taxonomy, systematics, phylogenomics, bioinformatics.

I confirm that I have read this submission and believe that I have an appropriate level of expertise to confirm that it is of an acceptable scientific standard.

# Reviewer report: Reviewer response for version 1

**DOI:** 10.21956/wellcomeopenres.26800.r126855

**Published:** 2025-07-26

**Authors:** Pedro Henrique Pezzi

**Affiliations:** 1 Biological Sciences, University of Arkansas Fayetteville, Fayetteville, Arkansas, USA

This paper generated a high-quality chromosome-level genome assembly of Andrena praecox, the small sallow mining bee, which represents a valuable resource for future research. The authors followed the standard Darwin Tree of Life pipeline, resulting in a genome with high BUSCO scores, contiguity and coverage. They also generated RNA data to help with genome annotation, making this a valuable resource for studies on bees.

Are sufficient details of methods and materials provided to allow replication by others?

Yes

Is the rationale for creating the dataset(s) clearly described?

Yes

Are the datasets clearly presented in a useable and accessible format?

Yes

Are the protocols appropriate and is the work technically sound?

Yes

Reviewer Expertise:

Phylogenetics, genomics, evolution, hybrization, population genetics.

I confirm that I have read this submission and believe that I have an appropriate level of expertise to confirm that it is of an acceptable scientific standard.
